# The role of multilevel factors in geographic differences in bicycle crash risk: a prospective cohort study

**DOI:** 10.1186/1476-069X-12-106

**Published:** 2013-12-09

**Authors:** Sandar Tin Tin, Alistair Woodward, Shanthi Ameratunga

**Affiliations:** 1 Section of Epidemiology and Biostatistics, School of Population Health, University of Auckland, Private Bag 92019, Auckland 1142, New Zealand

**Keywords:** Bicycling, Injury, Risk, Cohort studies, Mediation

## Abstract

**Background:**

Regular cycling plays an important role in increasing physical activity levels but raises safety concerns for many people. While cyclists bear a higher risk of injury than most other types of road users, the risk differs geographically. Auckland, New Zealand’s largest urban region, has a higher injury risk than the rest of the country. This paper identified underlying factors at individual, neighbourhood and environmental levels and assessed their relative contribution to this risk differential.

**Methods:**

The Taupo Bicycle Study involved 2590 adult cyclists recruited in 2006 and followed over a median period of 4.6 years through linkage to four national databases. The Auckland participants were compared with others in terms of baseline characteristics, crash outcomes and perceptions about environmental determinants of cycling. Cox regression modelling for repeated events was performed with multivariate adjustments.

**Results:**

Of the 2554 participants whose addresses could be mapped, 919 (36%) resided in Auckland. The Auckland participants were less likely to be Māori but more likely to be socioeconomically advantaged and reside in an urban area. They were less likely to cycle for commuting and off-road but more likely to cycle in the dark and in a bunch, use a road bike and use lights in the dark. They had a higher risk of on-road crashes (hazard ratio: 1.47; 95% CI: 1.22, 1.76), of which 53% (95% CI: 20%, 72%) was explained by baseline differences, particularly related to cycling off-road, in the dark and in a bunch and residing in urban areas. They were more concerned about traffic volume, speed and drivers’ behaviour.

**Conclusions:**

The excess crash risk in Auckland was explained by cycling patterns, urban residence and factors associated with the region’s car-dominated transport environment.

## Background

Using a bicycle, despite its proven health and other benefits [1-3], is rarely part of everyday travel in many countries due to concerns about traffic safety [4-7]. Cyclists generally bear a higher risk of injury than most other types of road users, per hour spent travelling [8,9] but the risk differs between and within countries [10-15]. This may be explained by geographic variations in population demographics, travel patterns, residential neighbourhoods and aspects of the physical environment. The ‘safety in numbers’ (or the ‘risk in scarcity’) effect is often cited as an explanation for a lower risk of injury in places with a higher level of cycling [10,11,14] but the relative contributions of this and other factors are poorly understood.

**Figure 1:**
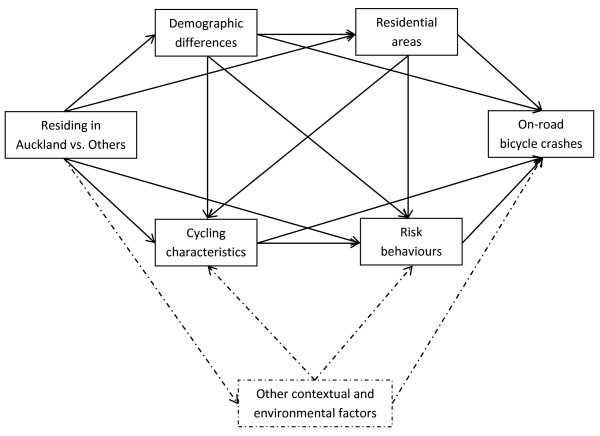
A simplified causal diagram depicting the role of mediating factors.

As in many other countries, cycling is an under-used mode of transport in New Zealand [16,17]. Travel patterns vary across the sixteen census regions [16,18] and so does the injury risk [14]. Auckland is the country’s largest and fastest growing metropolitan region accommodating one-third of the total population [19] (a map of New Zealand is provided in Additional file 1). The region also has the lowest level of active travel [16]. Consistent with the national statistics [14,20], our previous analysis of the data from the Taupo Bicycle Study shows that the risk of on-road bicycle crashes is higher in Auckland than the rest of the country [21]. This risk disparity may be mediated through multiple pathways involving differences in characteristics of the participating cyclists as well as broader contextual and environmental factors (Figure 1).

The Taupo Bicycle Study is a prospective cohort study designed to examine factors associated with regular cycling and injury risk. This paper used data from the study to assess the relative contribution of demographic, residential, cycling and behavioural risk factors in explaining the difference in crash risks between Auckland and the rest of the country, and to identify environmental factors that could play an important mediating role.

## Methods

### Design, setting and participants

The sampling frame comprised cyclists aged 16 years and over who enrolled online in the Lake Taupo Cycle Challenge, New Zealand’s largest mass cycling event held each November. Participants have varying degrees of cycling experience ranging from competitive sports cyclists and experienced social riders to relative novices of all ages.

**Figure 2:**
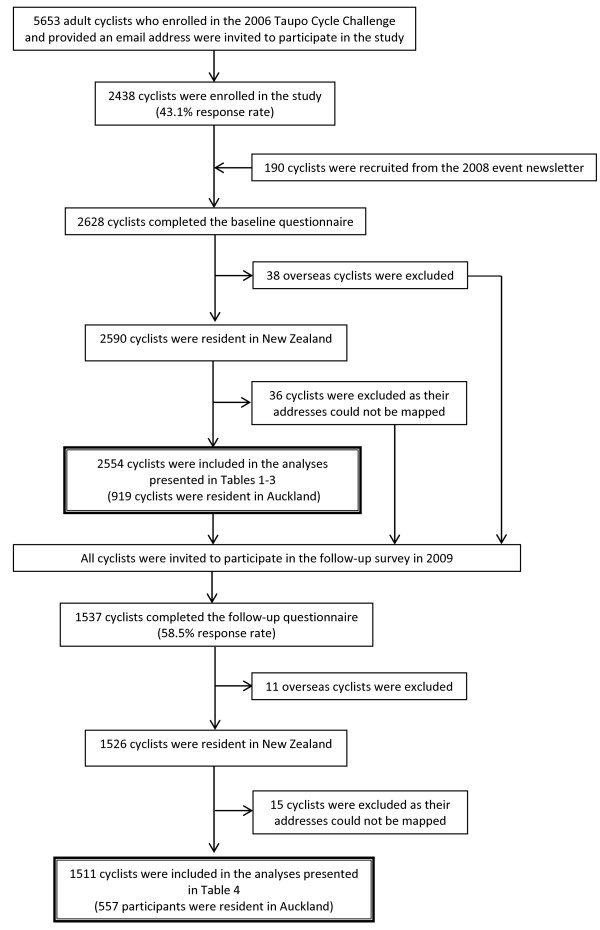
Flowchart of recruitment and follow-up of participants.

Recruitment was undertaken at the time of the 2006 event for the majority of participants, as described, in detail, elsewhere [22]. In brief, email invitations, containing a hyperlink to the study information page, were sent to 5653 contestants who provided their email addresses at registration for the event. Those who agreed to participate in the study were taken to the next page containing a web questionnaire and asked about demographic characteristics, general cycling activity and crash experience in the past twelve months and habitual risk behaviours with options ranging from ‘never’ to ‘always’. The questionnaire was completed and submitted by 2438 cyclists (43.1% response rate). Another 190 cyclists were recruited from the 2008 event by including a short description about the study in the event newsletter. All participants were resurveyed in December 2009 using a web questionnaire containing similar questions as the baseline questionnaire. The participants were also asked to rate the importance of specified factors that would influence cycling for transportation. A total of 1537 participants completed the questionnaire with options ranging from ‘not important at all’ to ‘very important’. Figure 2 presents the flow of study participation. Ethical approval was obtained from the University of Auckland Human Participants’ Ethics Committee.

### Crash outcome data

Crash outcome data were collected through record linkage to four administrative databases, covering the period from date of recruitment to 30 June 2011. All participants consented to link their data to the following databases.

In New Zealand, the Accident Compensation Corporation (ACC) provides personal injury cover for all residents and temporary visitors to New Zealand no matter who is at fault. The claims database is a major source of information on relatively minor injuries with over 80% of the claims relating to primary care (e.g., GPs, emergency room treatment) only [23]. Approval for record linkage was obtained from the ACC Research Ethics Committee.

The hospital discharge data contains information about inpatients and day patients discharged after a minimum stay of three hours from all public hospitals and over 90% of private hospitals in New Zealand [24]. The mortality data contains information about all deaths registered in the country [25]. Diagnoses in each hospital visit and underlying causes of death are coded under ICD-10-AM. Bicycle crashes were identified using the E codes V10-V19 and those that occurred on public roads were identified using the E codes V10-V18.3-9, V19.4-6, and V19.9. Readmissions were identified as described previously [26] and excluded.

In New Zealand, it is mandatory that any fatal or injury crash involving a collision with a motor vehicle on a public road be reported to the police. The crash analysis system data contains information on all police-reported bicycle collisions involving a motor vehicle.

For each participant, bicycle crashes identified across different databases were matched based on the date of crash allowing for a two-day difference, so as to avoid double-counting of the same crash.

### Analyses

The study sample was restricted to 2590 participants who were resident in New Zealand at recruitment. Participants’ addresses were aggregated into meshblocks, the smallest geographic units used by Statistics New Zealand. The meshblocks were then categorised as Auckland and other regions, and also as main urban areas (centres with populations of 30000 or more) and others [27]. To assess the degree of neighbourhood deprivation, the meshblocks were also mapped on to the 2006 New Zealand Deprivation Index (NZDep) [28] with decile ten the most deprived and decile one the least. This analysis excluded 36 participants whose addresses could not be mapped.

All analyses were performed using SAS (release 9.2, SAS Institute Inc., Cary, North Carolina). Baseline data were presented as means with standard deviations and medians with interquartile ranges for continuous variables and percentages for categorical variables. All the data were complete for 2435 participants (95.3%). Missing values were computed using multiple imputation with 25 complete datasets created by the Markov chain Monte Carlo method [29], incorporating all baseline covariates and injury outcomes. Crude and adjusted differences in baseline characteristics between the Auckland participants and the rest of the cohort were assessed using PROC GLM. In general, differences in residential characteristics were adjusted for demographic factors; differences in cycling characteristics were adjusted for demographic and residential factors; and differences in risk behaviours were adjusted for demographic, residential and cycling factors.

Bicycle crashes extracted through record linkage were categorised into on-road crashes (crashes that occurred on public roads) and others. As more than a single crash may be experienced during follow-up, incidence rates of repeated events were calculated separately for the Auckland participants and the rest of the cohort using the person-years approach. Confidence intervals were based on the Poisson distribution. The participants were censored on 30 June 2011 or date of death.

Cox proportional hazards regression modelling for repeated events were performed using a counting process approach to assess hazards of bicycle crash injury associated with residing in Auckland. Hazard ratios (HR) were sequentially adjusted for four domains of covariates: demographic, residential, cycling and behavioural risk factors (Figure 1). The mediating role of each domain was determined by the percentage reduction in the β coefficient after inclusion of each domain in the model using the approach described previously [30]: 100 × (β_crude_-β_adjusted_)/β_crude_. The 95% confidence intervals relating to each percentage attenuation were estimated using a nonparametric bootstrapping method with 2000 re-samplings (with replacement).

Finally, the data from the resurvey involving 1511 participants were used to compare perceptions of environmental factors that would influence cycling for transportation between the Auckland participants and others. For this analysis, the response options were dichotomised into ‘important’ (i.e., important and very important) and ‘not important’. Differences in perception were assessed using PROC GLM and adjusted for demographic, residential and cycling factors.

## Results

Of the 2554 participants who provided a complete New Zealand address at recruitment, 919 (36.0%) resided in the Auckland region. The Auckland participants did not differ from others by age and gender but were less likely to be Māori and more likely to be university graduates and reside in urban areas and least deprived neighbourhoods (Table 1). They had fewer years of cycling experience but cycled as much as others. They spent less time cycling off-road and more time cycling in the dark or in a bunch; and were less likely to commute by a bicycle. They were more likely to ride a road bike and use lights while riding in the dark but less likely to use reflective materials.

**Table 1:** Baseline characteristics of the participants in Auckland vs. the rest of New Zealand

**Baseline characteristics**		**Auckland (N = 919)**	**Others (N = 1635)**	**P-value**	
				**Crude**	**Adjusted**
Age	Mean (SD)	43.8 (10.6)	44.1 (10.3)	0.5	
Male	%	73.5	71.8	0.4	
Māori	%	2.5	5.0	0.003	
Education					
High school (secondary) or less	%	17.6	22.1	0.007	0.01^a^
Polytechnic	%	20.2	28.0	<0.0001	<0.0001^a^
University	%	61.9	49.6	<0.0001	<0.0001^a^
*Missing*	%	*0.2*	*0.2*		
Body mass index	Mean (SD)	25.2 (3.3)	25.4 (3.8)	0.1	0.3^a^
*Missing*	%	0.7	0.6		
NZDep 2006 score					
1–3	%	57.1	46.9	<0.0001	<0.0001^b^
4–7	%	32.9	37.7	0.01	0.03^b^
8–10	%	10.0	15.4	0.0001	0.0002^b^
Main urban area	%	95.5	69.4	<0.0001	<0.0001^b^
Years of cycling	Mean (SD)	6.4 (8.3)	7.2 (9.2)	0.02	0.006^c^
	Median (IQR)	3.0 (7.0)	3.0 (8.5)		
*Missing*	%	0.7	0.3		
Time spent cycling (hours per week)	Mean (SD)	5.8 (3.6)	5.7 (3.8)	0.3	0.4^c^
	Median (IQR)	5.0 (4.5)	5.0 (5.0)		
*Missing*	%	0.2	0.4		
% cycling off-road	Mean (SD)	5.8 (14.8)	9.8 (19.6)	<0.0001	<0.0001^c^
	Median (IQR)	0.0 (5.0)	0.0 (10.0)		
*Missing*	%	0.4	0.6		
% cycling in the dark	Mean (SD)	11.7 (16.4)	7.5 (12.0)	<0.0001	<0.0001^c^
	Median (IQR)	5.0 (20.0)	1.0 (10.0)		
*Missing*	%	0.2	0.2		
% cycling in a bunch	Mean (SD)	23.7 (28.1)	17.2 (23.2)	<0.0001	<0.0001^c^
	Median (IQR)	10.0 (45.0)	5.0 (25.0)		
*Missing*	%	0.8	0.8		
Cycle to work at least once a week	%	26.7	31.3	0.05	<0.0001^c^
*Missing*	%	*1.9*	*1.9*		
Mainly use road bike	%	89.5	85.0	0.001	0.0007^c^
*Missing*	%	*0.4*	*0.5*		
Crash in the past 12 months	%	32.5	29.9	0.2	0.9^c^
*Missing*	%	*0.2*	*0.2*		
Always wear helmet	%	98.6	98.7	1	0.5^d^
*Missing*	%	*0.4*	*0.4*		
Wear fluorescent colours					
Always	%	29.8	28.8	0.6	0.2^d^
Sometimes	%	49.5	51.3	0.4	0.4^d^
Never	%	19.7	19.1	0.7	0.7^d^
*Missing*	%	*1.0*	*0.7*		
Always use lights in the dark^e^	%	85.4	80.8	0.02	0.6^d^
*Missing*	%	*0.1*	*0.0*		
Use reflective materials in the dark^e^					
Always	%	44.9	51.7	0.006	0.02^d^
Sometimes	%	27.4	29.0	0.5	0.1^d^
Never	%	27.3	19.0	<0.0001	<0.0001^d^
*Missing*	%	*0.5*	*0.3*		
Ever listen to music while riding	%	16.3	16.3	0.9	0.3^d^
*Missing*	%	*0.8*	*0.4*		

^a^Adjusted for age, gender and ethnicity.

^b^Adjusted as above plus education and BMI.

^c^Adjusted as above plus NZDep 2006 scores and urban residence.

^d^Adjusted as above plus years of cycling, time spent cycling,% cycling off-road,% cycling in the dark,% cycling in a bunch, cycle to work, mainly use road bike and baseline crash history.

^e^Restricted to 1708 participants who reported cycling in the dark.

During a median follow-up of 4.6 years, 322 Auckland participants experienced 538 bicycle crashes, of which 337 occurred on public roads including one death due to a collision with a motor vehicle (Table 2). This corresponds to 133 crashes (95% CI: 121.93, 144.64), including 83 on-road crashes (95% CI: 74.55, 92.57), per 1000 person-years. The Auckland participants had a higher risk of on-road crashes (Crude HR 1.47; 95% CI: 1.22, 1.76) but had a similar risk of off-road crashes (Crude HR 0.96; 95% CI: 0.77, 1.21) compared to others.

**Table 2:** Bicycle crashes experienced during follow-up in Auckland vs. the rest of New Zealand

**Number of crashes experienced by each participant**	**All crashes**				**On-road crashes**				**Off-road crashes**			
	**Auckland**		**Others**		**Auckland**		**Others**		**Auckland**		**Others**	
	**N**	**%**	**N**	**%**	**N**	**%**	**N**	**%**	**N**	**%**	**N**	**%**
1	211	22.96	362	22.14	157	17.08	261	15.96	117	12.73	207	12.66
2	57	6.20	103	6.30	39	4.24	51	3.12	22	2.39	44	2.69
3	31	3.37	35	2.14	20	2.18	6	0.37	3	0.33	16	0.98
4	9	0.98	19	1.16	4	0.44	6	0.37	2	0.22	7	0.43
5	8	0.87	6	0.37	1	0.11	2	0.12	3	0.33	1	0.06
6	2	0.22	2	0.12	2	0.22			0	0.00		
7	1	0.11			0	0.00			0	0.00		
8	2	0.22			0	0.00			1	0.11		
9	1	0.11			1	0.11						
Total number of crashes	538		791		337		415		201		376	
Rate per 1000 person years	132.92		108.14		83.20		56.73		49.66		51.40	
(95% CI)	(121.93, 144.64)		(100.73, 115.94)		(74.55, 92.57)		(51.41, 62.46)		(43.03, 57.02)		(46.34, 56.87)	
Crude hazard ratio (95% CI)	1.23 (1.06, 1.42)				1.47 (1.22, 1.76)				0.96 (0.77, 1.21)			

The higher risk of on-road bicycle crashes in the Auckland participants was partly mediated by differences in baseline characteristics (Table 3). Demographic factors modestly attenuated the crude HR by 4% (95% CI: -1, 14). Subsequent adjustment for residential, cycling and behavioural risk factors resulted in a further 20%, 27% and 2% reduction in the HR. In particular, urban residence, time spent cycling off-road, time spent cycling in the dark and time spent cycling in a bunch contributed most. Overall, factors included in the fully adjusted model accounted for a 53% (95% CI: 20, 72) of the regional differential in crash risk.

**Table 3:** Risk of on-road bicycle crashes in Auckland vs. the rest of New Zealand with stepwise adjustments

**Models**	**Additional variables in the model**	**Beta estimates (SE)**	**Hazard ratios (95% CI)**	**% Attenuation (95% CI)**^**a**^
1. Unadjusted		0.382 (0.073)	1.47 (1.27, 1.69)	
2. Model 1 + demographics	Age	0.377 (0.073)	1.46 (1.26, 1.68)	
	Gender	0.375 (0.073)	1.46 (1.26, 1.68)	
	Ethnicity	0.370 (0.074)	1.45 (1.25, 1.67)	
	Education	0.369 (0.074)	1.45 (1.25, 1.67)	
	Body mass index	0.365 (0.074)	1.44 (1.25, 1.67)	4 (−1, 14)
3. Model 2 + residential factors	NZDep 2006 scores	0.368 (0.074)	1.44 (1.25, 1.67)	
	Urban residence	0.292 (0.077)	1.34 (1.15, 1.56)	24 (11, 49)
4. Model 3 + cycling characteristics	Years of cycling	0.291 (0.077)	1.34 (1.15, 1.56)	
	Time spent cycling	0.282 (0.077)	1.33 (1.14, 1.54)	
	% cycling off-road	0.245 (0.078)	1.28 (1.10, 1.49)	
	% cycling in the dark	0.217 (0.079)	1.24 (1.06, 1.45)	
	% cycling in a bunch	0.184 (0.079)	1.20 (1.03, 1.41)	
	Cycle to work	0.192 (0.079)	1.21 (1.04, 1.42)	
	Mainly use road bike	0.193 (0.080)	1.21 (1.04, 1.42)	
	Crash history	0.189 (0.080)	1.21 (1.03, 1.41)	51 (21, 74)
5. Model 4 + risk behaviours	Use helmet	0.188 (0.080)	1.21 (1.03, 1.41)	
	Use fluorescent colours	0.188 (0.079)	1.21 (1.03, 1.41)	
	Use lights in the dark	0.189 (0.079)	1.21 (1.03, 1.41)	
	Use reflective materials in the dark	0.179 (0.080)	1.20 (1.02, 1.40)	
	Listen to music while riding	0.179 (0.080)	1.20 (1.02, 1.40)	53 (20, 72)

^a^95% bootstrap confidence interval.

In addition, there were regional differences in participants’ perceptions of environmental factors likely to influence cycling for transportation (Table 4). The Auckland participants were more likely to report ‘driver attitude and behaviour’, ‘road safety’, ‘traffic en route’, ‘need a car for other reasons (e.g., school run)’, ‘breathing polluted air’ and ‘personal security’ as important factors that would prevent cycling for transportation. They were also more likely to report ‘more bike lanes’, ‘changing driver attitude and behaviour’, ‘reduced vehicle speed’ and ‘bike friendly public transport’ but were less likely to report ‘rising costs of fuel’ as important factors that would encourage cycling for transportation. Moreover, they were more likely to report ‘showers’ and ‘charges for car parking’ as important factors that would encourage cycle commuting.

**Table 4:** Environmental factors perceived as important in influencing cycling for transportation in Auckland vs. the rest of New Zealand

**Environmental factors**	**Auckland (%) N = 557**	**Others (%) N = 954**	**P-value**	
			**Crude**	**Adjusted**^**a**^
***Barriers to cycling for transportation***				
Adverse weather	82.6	82.4	0.9	1
Too hilly	20.1	24.5	0.05	0.1
Would take too long	49.2	50.9	0.5	0.9
Too far	41.3	42.5	0.7	0.7
Too short a distance to use a bike	26.4	23.4	0.2	0.8
Personal security	50.1	38.4	<0.0001	0.0002
Road safety	79.9	69.2	<0.0001	<0.0001
Traffic en route	79.0	67.3	<0.0001	<0.0001
Driver attitude and behaviour	80.8	71.5	<0.0001	<0.0001
Availability of other easier transport	42.0	38.5	0.2	0.3
Need a car for other reasons (e.g., school run)	72.9	67.6	0.03	0.01
Breathing polluted air	50.1	41.3	0.0009	0.009
***Enablers of cycling for transportation***				
More bike lanes	91.4	87.4	0.02	0.04
More bike paths	78.5	80.3	0.4	1
Need to negotiate fewer difficult intersections	75.9	70.7	0.03	0.1
Better road conditions	84.0	81.5	0.2	0.5
Reduced vehicle speed	63.7	58.8	0.06	0.03
Changing driver attitude and behaviour	90.1	84.7	0.003	0.01
Bike friendly public transport	52.1	39.5	<0.0001	<0.0001
Secure bike parking in public places	69.3	73.3	0.1	0.3
Bike designed for transportation	26.6	29.0	0.3	0.07
Car parking restrictions	32.1	32.2	1	0.7
Rising costs of petrol	41.3	48.3	0.008	0.03
***Enablers of cycling to work/education***^***b***^				
Showers	94.1	88.8	0.002	0.03
Lockers and changing facilities	90.6	84.7	0.004	0.1
Secure bike parking	91.9	86.7	0.006	0.1
Pool bikes	6.2	7.6	0.3	0.1
Financial assistance to buy a commuter bike	10.3	9.7	0.7	0.4
Bike maintenance classes/facilities	15.2	15.5	0.9	0.4
A more flexible dress code	29.5	29.0	0.9	0.9
More flexible working hours	38.5	40.2	0.6	0.6
Charges for car parking	25.5	19.1	0.009	0.04

^a^Adjusted for demographic, residence and cycling factors.

^b^Restricted to participants who reported travelling to work/education at least once a week (N = 455 in Auckland and N = 734 in the rest of New Zealand).

## Discussion

### Main findings

In this study, the risk of on-road bicycle crashes was 47% higher for participants who were resident in the Auckland region at recruitment compared to others. Approximately 53% of the excess risk was attributed to differences specifically examined in the study, particularly cycling off-road, in the dark and in a bunch and urban residence. Differences in Auckland and non-Auckland participants’ perception of environmental determinants of cycling relating to other dangers of the traffic environment suggest other factors that could explain the remaining difference in crash risk.

### Strengths and limitations

In this prospective cohort study, baseline data were near-complete as mandatory fields and validation checks were incorporated in the web questionnaire. Crash outcome data were collected from four administrative databases, thereby minimising potential biases associated with loss to follow-up and self-reports.

This analysis, however, excludes minor crashes not coming to the attention of the police or medical personnel, which amounts to more than two-thirds of self-reported crashes in this study [31]. Ascertainment of crash outcomes may be affected by personal, social and health service factors [32] as well as the quality of individual data sources [33-35] and record linkage [36]. Nevertheless, our risk estimates are likely to be conservative as potential misclassification of crash outcomes may be non-differential in a prospective cohort study [37]. Self-reported exposure data may not be accurate and may change over time. In particular, migration may have occurred during follow-up. In the follow-up survey, however, only 1.1% of the Auckland participants reported moving to other regions and 1.3% reported moving overseas, and 0.3% of the participants from other regions moved to Auckland and 0.7% reported moving overseas. In fact, misclassification of the mediating variables is more important than that of the exposure (due to migration) and may underestimate the% attenuation presented in this paper [38]. Finally, our participants cannot be considered representative of all New Zealand cyclists; however, this may have minimal impact on the risk estimates [39]. Importantly, the participants represented a wide variation with regard to demographics, cycling exposure and experience.

### Interpretation

There were significant differences in demographic, residential, cycling and some behavioural characteristics between the Auckland participants and the rest of the cohort. Similar demographic differences were observed in the general population (see Additional file 2). Moreover, compared to the national average, more people use a car and less use active transport modes in Auckland [16,40] despite its favourable climate [41]. The danger of the region’s car dominated road environment is reflected in the participants’ perceptions about the determinants of cycling. Consequently, the Auckland participants had a 47% higher risk of on-road bicycle crashes but had a similar risk of off-road crashes compared to others in the study.

Differences examined in this study accounted for 53% of the excess risk of on-road crashes in Auckland. The Auckland participants were less likely to be Māori and more likely to be socioeconomically advantaged, reflecting demographic differences observed in the general population (see Additional file 2). Demographic differences exist in the risk of bicycle-related injuries [8,21], but accounted for only 4% of the regional disparity in crash risk in this study. Urban residence, however, explained an additional 20% of the risk disparity. Of note, Auckland is New Zealand’s largest urban region and its population density is more than double that of any other region. Previous studies show that the majority of on-road bicycle crashes occur in urban areas [42,43] where there are more people, heavy traffic and busy intersections while more severe crashes tend to be located in more remote areas where vehicles tend to travel at higher speeds [43-45]. However, it was not possible to differentiate crashes in terms of severity of injury in this study as multiple administrative databases were used.

Differences in cycling characteristics contributed to a further 27% attenuation in the hazard ratio. The greatest contribution came from less time spent cycling off-road and more time spent cycling in the dark and in a bunch by the Auckland participants. The former two may increase exposure to the crash risk but bunch riding has also been associated with a higher risk [21]. It is possible that cyclists are more likely to take risks [46] and less likely to notice road hazards [47] while riding in a group. Other cycling characteristics and behavioural factors such as use of visibility aids and distraction contributed modestly to regional differences in crash risk.

After all baseline differences were taken into account, the risk of on-road crashes was still 20% higher among the Auckland cyclists. This may be explained by other contextual and environmental factors. While the climate may influence cycle volume [48] and safety [49], Auckland’s relatively temperate climate is unlikely to be particularly hazardous. Similarly, with respect to topography, many parts of New Zealand are hillier than Auckland (see Additional file 1). Given the similarity in the risk of off-road crashes between Auckland and other regions, differences in travel patterns and traffic environment are more likely to contribute to the remaining disparity in crash risk.

Auckland is characterised by low density urban growth and an automobile-centred transportation system, encouraging car dependency, traffic congestion and air pollution [50,51]. This may pose risks to its users [52], particularly those who are vulnerable. Previous research on the ‘safety in numbers’ effect associated a higher risk of bicycle crashes with a lower level of cycling [10,11]. This is true in the New Zealand context particularly if the level of car use is also taken into account [14]. Moreover, a car-dominated road environment tends to discourage people from engaging in active travel [53]. In this study, the Auckland cyclists had more concerns about driver attitude and behaviour, traffic en route, difficult intersections, vehicle speed, road safety and polluted air, concerns which may be more prevalent in the general population [54].

## Conclusions

The risk of on-road bicycle crashes was higher in Auckland than the rest of New Zealand. Approximately half of the excess risk was contributed by differences examined in this study particularly related to cycling patterns and urban residence. The remaining difference in crash risk may be explained by factors generally associated with the region’s car-dominated transport environment. This underscores the need for cooperative efforts to promote cycling and cyclists’ safety in the Auckland region.

## Abbreviations

ACC: Accident compensation corporation; CI: Confidence interval; HR: Hazard ratio; ICD: International classification of diseases; NZDep: New Zealand deprivation index.

## Competing interests

The authors declare that they have no competing interests.

## Authors’ contributions

STT contributed to the conception and design of the study, acquisition, analysis and interpretation of data and drafting of the manuscript. AW and SA contributed to the conception and design of the study, interpretation of data and revision of the manuscript. All authors read and approved the final manuscript.

## Supplementary Material

Additional file 1 **Map of New Zealand.** The boundary for the Auckland region is marked in red. Click here for file

Additional file 2 Characteristics of Auckland vs. New Zealand. Click here for file
