# Supplementary figures and images for: The role of multilevel factors in geographic differences in bicycle crash risk: a prospective cohort study
Source: Environ Health. 2013 Dec 9;12:106. doi: 10.1186/1476-069X-12-106 (PMC3893370; doi:10.1186/1476-069X-12-106)

## Map of New Zealand


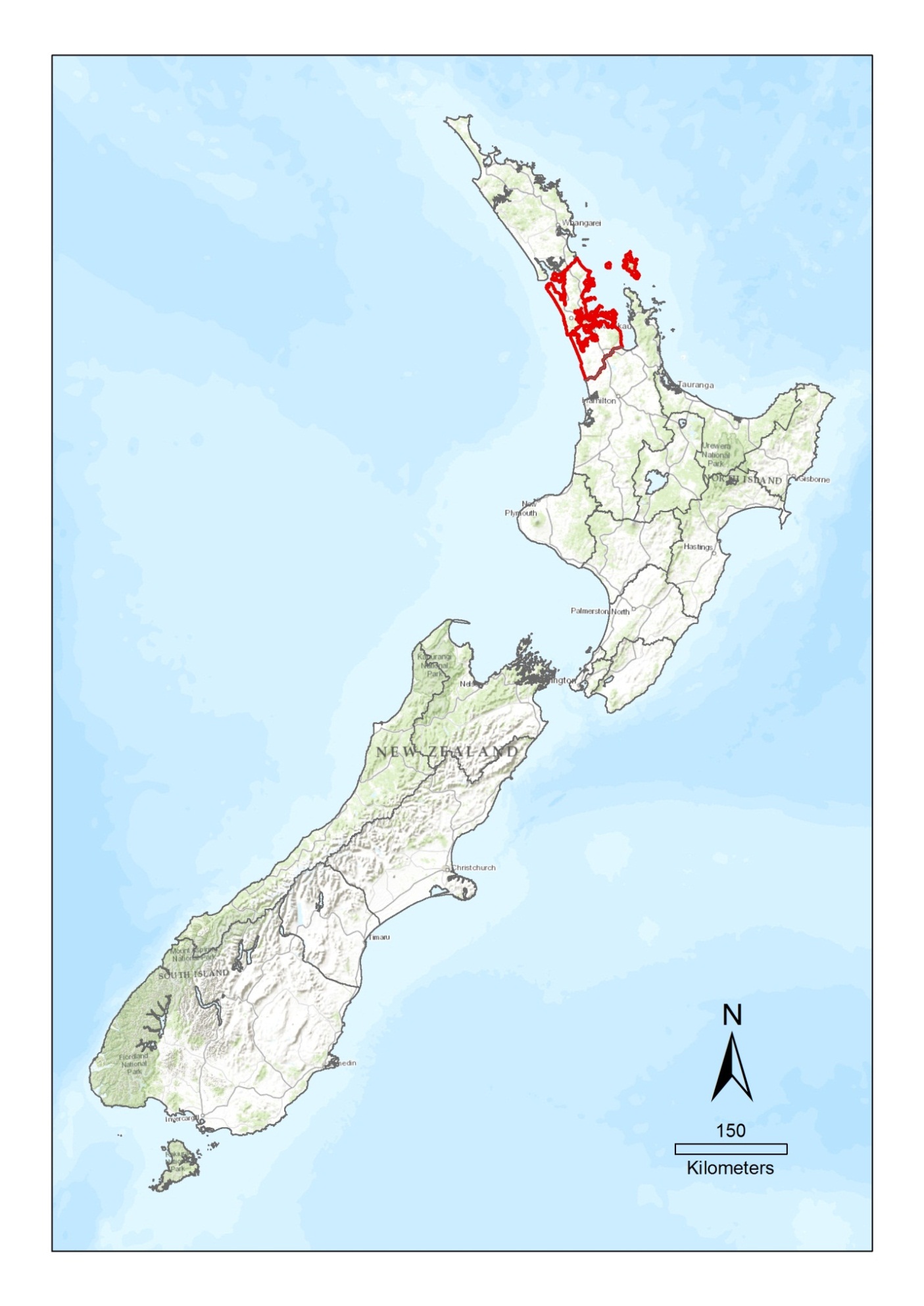


The boundary for the Auckland region is marked in red.

Supplement: Additional file 1 — Map of New Zealand. The boundary for the Auckland region is marked in red. [file 1476-069X-12-106-S1.docx]
