# Supplementary material for: The role of multilevel factors in geographic differences in bicycle crash risk: a prospective cohort study
Source: Environ Health. 2013 Dec 9;12:106. doi: 10.1186/1476-069X-12-106 (PMC3893370; doi:10.1186/1476-069X-12-106)
Supplement: Additional file 2 — Characteristics of Auckland vs. New Zealand. [file 1476-069X-12-106-S2.docx]

## Characteristics of Auckland vs. New Zealand

| **Characteristics** | **Auckland** | **New Zealand** |
| --- | --- | --- |
| **Population density** (per km^2^)^1^ (2006) | 215.3 | 14.9 |
| **Demographics**^1^ (2006) |  |  |
| Median age (years) | 33.9 | 35.9 |
| Male | 48.7% | 48.8% |
| Maori | 11.1% | 14.6% |
| Post-school qualification | 42.5% | 39.9% |
| Median annual personal income | $26,800 | $24,400 |
| **Transport** |  |  |
| Trip distance^2^ (km) (2003-8) |  |  |
| Mean (SD) | 7.8 (5.6) | 8.3 (4.3) |
| Median (IQR) | 3.3 (6.9) | 2.9 (6.5) |
| Hours spent travelling per person per year |  |  |
| (% of total travel time)^2^ (2006-9) |  |  |
| Car/van driver | 223 (54%) | 194 (51%) |
| Car/van passenger | 107 (26%) | 103 (27%) |
| Cyclist | 2 (1%) | 6 (2%) |
| Pedestrian | 50 (12%) | 50 (13%) |
| Public transport (bus, train, ferry) | 18 (4%) | 15 (4%) |
| Main means of travel to work^1^ (2006) |  |  |
| Car/van/truck driver | 79.6% | 77.0% |
| Car/van/truck passenger | 5.8% | 6.0% |
| Cyclist | 1.0% | 2.5% |
| Pedestrian | 4.9% | 7.0% |
| Public transport (bus, train) | 6.9% | 5.2% |
| **Climate summary**^3^ (1971-2000) |  |  |
| Mean annual rainfall (mm) | 1240 | 1419 |
| Mean annual sunshine (hours) | 2060 | 1988 |
| Mean temperature (ºC) | 15.1 | 12.3 |

1 – Source: 2006 Census. Statistics New Zealand

2 – Source: Household Travel Survey data. Ministry of Transport

3 – Historical averages for the main cities/centres. Source: NIWA National Climate Database. National Institute of Water & Atmospheric Research
